# Secondary Batteries and the So-called Storage of Electricity*Read before the Chicago Electrical Society, Jan. 15, 1883.

**Published:** 1883-02

**Authors:** Roswell Park

**Affiliations:** Surgeon to the Michael Reese Hospital; Lecturer on Surgery in Rush Medical College; President of the Chicago Electrical Society; 1558 Wabash Ave.


### ARTICLE III.

### SECONDARY BATTERIES AND THE SO-CALLED STORAGE OF ELECTRICITY.* By ROSWELL PARK, A.M., M.D., Surgeon to the Michael Reese Hospital; Lecturer on Surgery in Rush Medical College; President of the Chicago Electrical Society.

It is but a comparatively short time since *amateurs* in general and electrical science in this country, began to hear of the so-called "accumulation of electricity" and of "storage batteries." They have been much longer known, of course, to professional electricians, many of whom have devoted to the subject a great deal of patient thought and study. As the matter stands to-day it is perhaps the most important topic at present before the electrical world, and as such is an eminently proper one for discussion in this Society. It is my purpose, then, to-night, to go briefly over the ground, giving a short account of the steps by which our present position was reached and a general idea of the *modus operandi* of secondary batteries, their construction, and of some most recent American improvements and patents covering ingenious applications of the same to actual work.

To sum up the whole idea in brief compass, it consists in storing up energy, in converting actual or potential energy into a latent form, and then releasing it again in active operation. It is well known that by proper methods any one kind of force may be converted into any other. In the ordinary galvanic battery molecular attraction or chemical energy is correlated into that

* Read before the Chicago Electrical Society, Jan. 15, 1883.

form of vibratory motion known as electricity. In the "storage battery" this same process takes place only at the expense of several correlations of the particular force originally resorted to.

Let us take the simplest form of secondary battery,—two lead plates separated from each other, each connected with one pole of a galvanic battery, say a Grenet cell, both plates immersed in water acidulated with 10 per cent. of sulphuric acid,—and study what takes place. With the passage of the current electrolytic action occurs, the acidulated water is decomposed, and the oxygen thus given off goes to the + pole or plate, which thus becomes coated with a film of plumbic oxide. When this film is thick enough the cell is in a condition to give some result. Let us now disconnect this element from the Grenet cell, and simply join the wires connected with the two lead plates. The action is then, so to speak, reversed, as follows: The metallic lead in the plate connected with the — pole, which was not covered with oxide, now acts like the zinc plate in an ordinary battery, partly giving up all the hydrogen which it has absorbed while the water was decomposing, and partly combining with the acid in the water, forming plumbic sulphate. The hydrogen thus released, and that from the acid which has been replaced by lead, now unite with the oxygen of the plumbic oxide coating the other plate, reducing it to a lower oxide, part of which lower oxide is also converted into a sulphate. When the plates are again charged the effect of the current is to convert the lead sulphate on one plate into spongy metallic lead, and that on the other into peroxide.

To state the action in different terms, it is this: Chemical action in the Grenet cell is converted into electrical energy and as such is sent into the secondary battery where, by conversion, it again becomes chemical energy, producing the changes above specified. It is this chemical energy which is stored up, and not electricity. When the wires or poles are again joined it is once more converted into electricity, ready to do any kind of work and to be transposed into heat or some form of motion.

The advantage of all this obtains just here, that this second correlation of force can be almost indefinitely postponed and commanded at will. The potential energy stored up in the secondary battery, *minus* a certain loss, can be used or drawn upon days or even weeks after it was accumulated, though the longer this be postponed the greater the loss. Hence, the term "storage of electricity," which we see to be erroneous, as well as the term "storage battery." The strictly proper term to use, then, is "polarization," or "secondary" battery, instead of "accumulator;" though I doubt whether the phrase "storing electricity" will soon pass out of common use. The secondary current is of comparatively brief duration unless resistance be introduced, but its total energy is nearly equal to the total amount received from the charging source. Inasmuch as it is brief it will be much stronger while it lasts than the primary or battery current. How advantage is taken of this fact shall appear further along.

Knowing now what a secondary battery fully consists of, *in its simplest shape*, we can more intelligently go over a few points in the history of its development.*

Quoting partly from Morton's paper, I would mention the following facts: Gautherot noticed in 1801 that the platinum or silver wire which had been used for terminals of a battery doing electrolytic work could yield a brief galvanic current without the aid of a battery. Ritter, of Jena, in 1803 confirmed this observation, and even made what he called a "secondary pile" of discs of copper and moist paper, alternately placed. This, in a small way, had all the properties of one of our modern instruments. He experimented with many metals. Marianini, of Vienna, corrected the error of those observers who had regarded all such "reservoirs" as they would Leyden jars, and showed that this power was due to polarization.

Humphry Davy, De la Rive, and Farraday included the subject in their researches, but Grove, in 1839, made the most positive advance by showing that how a constant current was kept up for days, when two glass tubes, one containing oxygen, the other hydrogen, were lowered over the two submerged ends of a platinum wire circuit. This arrangement was known as his "gas battery." If in tubes properly arranged to form such a

* Those desiring greater detail are referred to Morton's readable paper in *Harper's Magazine*, Dec. 1882, which contains numerous references to the bibliography of the subject. Also to general works on Electricity, especially to Planté's.

battery the gases be first generated by electrolytic action, or, much better still, if in each tube be hung a piece of platinum foil, covered with spongy platinum, and connected by wires not submerged, and these tubes then filled with gases as before, a counter-current will be generated as soon as the original is stopped, and last as long as any gases are exposed to the action of the spongy platinum. Grove constructed a battery of fifty such cells, and produced effects precisely similar to those we now get, only much weaker.

Many others among original workers have devoted much time to the development of the experience thus gained, among them Wheatstone, Schoenbein, Poggendorf, Becquerel, Du Moncel, Planté, Du Bois, Reymond, Thomson, Varley, Tait, and numerous others. But no one has done as much as M. Gaston Planté in studying the principles involved, and elucidating them for others. To him, perhaps, more than to all the rest combined, are we indebted for what we know of the matter to-day.

In 1879, Planté published his researches (*Recherches sur l'Electricité*), in which he goes over the ground most thoroughly. Planté's simple cell is the one which I have already described. The great drawback in the use of such cells is the length of time it takes to oxydize the lead, and convert it into that spongy form, in which it gives the best results. Lead does not oxydize rapidly, but requires months, or years, even, in acidulated water, and until there is, when charged, a good thick coating of oxide on one plate, and of spongy lead on the other, our cell will be a disappointment, while all the lead not so converted is, literally, a dead weight.

To *form* such a battery more speedily, that is, to put its lead plates in the best condition, Planté adopted this method: He found two or three Bunsen or Grove cells more effective than a large number of ordinary sulphate of copper cells. The first day the secondary battery is charged, alternately, in opposite directions, five or six times, and discharged after each reversal, the charges in each direction lasting, respectively, fifteen, thirty and sixty minutes. The last charge is allowed to remain until the next day, when the process is repeated, but each charge lasting two hours. The element is then left at rest, charged, for eight days, after which the charge is reversed and allowed to remain fifteen days; then, for one month, two months, etc., the power still increasing, until the whole thickness of the lead plates is involved in the alternating chemical processes. The intervals of rest allow the crystalline structures of the lead time to harden before reversal of the current.

I had, in August last, the pleasure of going over more or less of this ground with M. Planté in his own laboratory, and was delighted with his results. He showed me a secondary battery of 840 test-tube cells, each with its two tiny lead plates, the whole arrangement being some years old. These were so arranged that they could be coupled either for quantity or tension. When charged side by side, and discharged in series, the differences of potential were very great. With half this battery, (420 cells), thus charged from two Bunsen cells for a very few seconds, I saw him obtain all the effects of static electricity. He also showed me a very small secondary battery, not more than ten centimetres high, which he had had in use for several years. Connecting this for twelve seconds with the two Bunsen cells, and removing it, he was able to so heat a platinum wire as to light a taper twelve times. He also demonstrated to me what he calls the "residual charge" after the element has been apparently completely discharged. In fact, in the course of a day this residual charge can be shown a number of times in one cell. What follows will serve to explain this fact.

Before going on to further consider the work of others, this seems a suitable time to note more exactly the chemistry of the changes which take place during the alternate processes of charging and discharging. The oxides of lead vary in their conducting power, the highest oxide being the best conductor. When reduction of this oxide occurs, by discharge of the battery, some of the oxygen given off undoubtedly goes to help oxydize the remaining metallic lead. This fresh lead doubtless in time assumes the same degree of oxidation as the rest, but very little of the peroxide is ever reduced to the lowest or protoxide, or it would be eagerly attacked by the sulphuric acid. The lower oxides formed by reduction are of little use for battery purposes, and their resistance is much greater; hence, the gradual loss of battery power on allowing a charged battery to stard. Hence, also, the reason that the oxygen plate constantly increases in capacity; and, hence, too, the residual charge.

Most of the hydrogen is either absorbed by the hydrogen plate, especially when the lead is in a spongy condition, or, as some hold, it enters into very feeble combination with it. If at any time the current be reversed from the usual order, the coating of oxide will be fully reduced by the nascent hydrogen evolved on it, and a coating of porous lead will be left. Hence, the benefit of alternating the current in "forming" a cell. This porous metal is in a much better condition to absorb and retain hydrogen. After the process of forming is complete the hydrogen plate does not increase in capacity as does the oxygen plate.

In ordinary descriptions of storage apparatus enough importance has not been attached to the varying degrees of resistance in the coatings of the lead plates. Indeed, I am not sure that the subject has been fully investigated until lately. Brush has pointed out how barriers to the free passage of the electric currents are formed, as not only the peroxide is reduced by the combination of hydrogen and oxygen *within* the mass of peroxide, thus generating water which is a poor conductor, but also by the same production of water the pores of the spongy lead on the hydrogen plate.

A great detriment to the successful operation of polarization batteries in this: the coating of peroxide on the oxygen plate occupies more space than did the original lead. This shell must expand in forming, and evidently will have a different co-efficient of expansion. The tendency then is for this coating to crack. If one side of a plain lead surface be varnished and the other side be oxydized, the side acted on becomes convex; when the oxide is reduced the plate becomes concave. This must exert a disintegrating action on the coating and cannot but be detrimental. Finally, the coating is liable to peel off, or to be detached by liberation of gases when the charging current is too strong. Moreover, a portion of lead sulphate is formed whenever the oxide is too much reduced; this occupies more space than the oxide and increases the above effect; it besides, fills up the pores of the spongy mass, and is a bad conductor.

It will thus be seen that in the secondary battery of the future many obstacles are to be overcome. How far they have been surmounted in that of the present is a part of the subject now to be dealt with.

Camille Faure has studied how to "form" the elements more speedily than can be done by Planté's process, and how to reduce the dead weight,—a double problem. Planté had combined a number of plates in condenser shape, or had rolled them up concentrically, but depended on the slow process to oxydize them. Faure made the happy discovery that he could quickly prepare them by covering the lead plates with a paste of red oxide and sulphuric acid, and then rolling them up, separated from each other by strips of rubber or sheets of felt kept in place by lead rivets. This red lead is largely converted into a sulphate, and this on the first action of the charging current reduced, as before, on the one plate into spongy lead, on the other into peroxide. Faure's batteries are now often made of numerous flat plates, properly connected, covered with the paste and then insulated from each other by some woven fabric, and the whole packed in a rectangular box filled with dilute acid. However arranged, when the charging current ceases there results a condition of unstable equilibrium. When the poles are connected a vigorous current in the opposite direction is the result.

Still later, and within the year past, Faure has guarded by U. S. Letters Patent (252,002), another improvement. The electrodes are made not by formation of a spongy layer as a part of the original plain, but by the addition of a layer of active material—which may be of metal, metallic oxide or salt—which layer is capable of becoming porous or spongy—to suitable plates or supports which latter may be of non-metallic substance. This layer may be deposited with paste, cement, by galvanic action, chemical precipitation, or otherwise. In order to render the active layer more porous inert material may be mixed up with it; for example, crushed coke. It is retained by an open-work or porous medium, which allows free percolation of electrolytic fluids, but prevents separation by jarring. The retaining medium may be felt, asbestos paper, netting or gauze of cane, gutta-percha, or any suitable material. The whole space between the electrodes may be occupied by porous material. In place of dilute sulphuric acid a solution of any salt will answer.

Prior to these last patents of Faure's, Houston and Thomson devised a different form of battery. In a proper vessel a plate of copper is suspended near the bottom. The cell is filled with solution of zinc sulphate. Near the top is a plate of carbon; the two plates being separated by a porous diaphragm. The action of the charging current is to decompose the solution, the zinc being deposited in metallic state upon the carbon. The liberated sulphuric acid attacking the copper forms cupric sulphate, whose solution remains at the bottom on account of its gravity. Connect the two poles of this cell and we have the conditions of a Callaud or gravity cell, a current being at once generated. It is an ingenious though not powerful battery.

D'Arsonval hit upon nearly the same plan at about the same time. De Pezzer claimed very great advantage for extremely thin plates of lead; but the reason for this was self-evident. De Meritens modified previous plans by folding his lead plate like the leaves of a book, and then, as it were, interleaving them. Kabadt, in order to increase surface and decrease weight, figured his plates with perforations. Ayrton proposed to decompose the acidulated water under pressure, and to utilize the extra hydrogen given off.

Rousse has constructed a very ingenious cell, his object being to employ those metals which best absorb gases. To this end he inserts in the acidulated water a plate of lead which shall be the oxygen pole, and a plate of palladium, which absorbs 900 times its volume of hydrogen. This cell is very powerful. He makes another, with one plate lead, as before, and the other of sheet iron, which absorbs 200 vols. of hydrogen—placing both in a fifty per cent. solution of ammonium sulphate.

Lastly, Maiche re-charges ordinary cells, when exhausted, by placing them in the current of an ordinary dynamo, it being well known that Leclanché cells, for example, can be thus recharged.

None of these devices, however, with the possible exception of the palladium cell, excel a well-formed Planté element. To turn now to what American talent has lately accomplished in this direction, the following claims or specifications of patents are certainly worth notice:

Brush patents (263,756) an alloy of lead with a non-oxidizable substance, upon which an active absorptive coating is applied. Another patent of his (264,211) covers a device for placing in a bath numerous plates or elements of lead, connected with the oxygen pole of a generator and a single electrode of lead or copper connected with the hydrogen pole, by which the former are all charged, or coated with oxide. They can then be removed, and placed in individual cells, all ready to charge and discharge. One action then suffices to reduce into spongy lead the coating on the plate to be used as the hydrogen plate, and to assist in the formation of the coating on the other. He has further patented (266,090) a method of ribbing the plates, so that they shall better hold the coating they are to receive, and expose a larger surface.

Eaton's patent (266,114) covers the treatment of skeleton plates in a solution of metallic salts, as, for instance, plumbic acetate, in the presence of zinc, by which, the zinc having a greater affinity for the acetic acid than the lead, the latter is obtained in spongy form upon the plates. These plates are then coupled up as usual to form the battery.

W. A. Shaw's patent (266,262) includes these improvements: an addition to the lead, or lead oxide of sulphur, and the preparation of the two into a paste with sulphuric acid; a solid compound or salt which is soluble, and whose solution is an electrolytic fluid, as a filling between the electrodes, when mixed with some porous material; and a special preparation of the electrodes with potassic nitrate or similar salt, to render them more active. For this latter purpose, they are dipped into a saturated solution of saltpeter, removed to dry, and then repeatedly dipped and dried; all of which gives them a sort of glaze. He further makes the electrodes tubular, so that a current of cold air may be passed through them while they are being charged, to overcome elevation of temperature. Twenty parts of red lead to one of fine sulphur, or equal parts of white lead and sulphur, used as a coating, give good results. For instance, if a coiled or serpentine tube be used as an electrode, this coating is pasted into all the interstices before being finally glazed. For porous filling, sand or sawdust, mixed with any of the alkaline nitrates, or zinc sulphate, or various other salts, may be employed. Shaw has also discovered that an alloy of bismuth and lead, say 1-10 of one per cent. bismuth, is an improvement on pure lead. He makes all his tubular electrodes with a core of pure lead and an outer surface of this alloy.

Blanchard's plan (267,138, is for concentric leaden cylinders, with perforated tubes in annular spaces between, said cylinders being so raised as to give outlet below, and a filling of granulated lead or oxide of lead packed into the annular spaces.

Starr (267,275) would mix finely divided active material, as red lead, with a non-active material, which, being brought into a plastic or fluid condition, sets or hardens in a porous condition without artificial heat. Finney's patent (267,860) covers the use of leafy, flaky or fibrinous lead.

Starr has taken out another patent (268,308) later than Faure's, which differs from it in this respect: the active material applied to the plate is mixed with fiber or filaments, in contradistinction to pulverized coke or sawdust, whereby the porous mass is held more securely. Each plate electrode is provided with plates extending through it, for escape of surplus gas, and is separated from the others by a porous partition. At the top of the case containing the whole is an automatic valve, worked by an electro-magnet, for surplus gas to escape, and regulated by the current.

Eaton, also, has very recently patented (268,360) an element somewhat resembling that of Houston and Thompson. His combination is of an electro-negative element of spongy lead, a solution of cupric sulphate, and a positive element of carbon, or carbon and metal, in a finely divided state and loose.

After this consideration of the construction and working details of secondary batteries, the very important question arises, as to what their capabilities are. The statement has already been made, that there was a certain loss. What is the amount of this loss?

MM. Tresca, Allard, Le Blanc, Jubert and Pottier, made a report to the French Academy, whose main points may be thus summarized: (*Parville*, "*L'Electricité et ses Applications.*") A battery of 35 secondary cells, weighing each 43 kilos., in all about one and a half long tons, was charged by a Siemens dynamo of one and one-half horse power for nearly 23 hours; or equal to one horse power for 35 hours. Of this power, 34 per cent. was sheer waste in overcoming friction and in useless work, while 66 per cent. was sent into the battery. Of the full amount sent into the battery, 60 per cent. was recovered in the form of electricity. In other words. of 35 hours' work into a ton and a half of storage battery, 14 hours' work could be made actually available; or enough, as Morton puts it, to run twelve of Edison's eight-candle lamps for fourteen hours. Or, to put it in yet another shape, there were 35 cells, and 35 hours of one horse power expended, that is, a horse power for each cell. Each cell received 270,000 kilogrammeters of energy, of which it yielded in return 40 per cent., or 108,000. Each kilogram of lead thus gave up 2,500 kilogrammeters of actual force.

But since this investigation, much better results have been achieved. M. Planté has measured the amount of working force returned from some of his best formed elements, and found it to be 88 to 89 per cent. Hospitallier also has had better results. Gladstone and Tribe have carefully studied the causes of loss, which they find to be local action between the lead plate and the peroxide deposited on it—already alluded to—and the resistance of the lead compounds to the passage of the current, by which energy is correlated into other than the desired form. By properly adapting the constituents and regulating the discharge, and by "resting" the battery, Ayrton and Perry have recovered 82 per cent. of power put into these cells.

These latter results are far better than those yielded by the experiments of the French committee, and are an indication of the amount of energy we may legitimately expect these secondary batteries to yield back. I have no doubt that the battery of the near future will steadily yield 80 per cent. of energy sent into it.

General interest in the subject may justify me in concluding with an exceedingly brief resumé of a very few of the recent United States patents, covering applications of secondary batteries, which seem to deserve notice on account of their ingenuity or serviceability.

T. A. Edison (Pat. 218,167), makes the statement that when a secondary battery is fully charged decomposition of the liquid commences, and gases are developed. He arranges two secondary elements so that one may be charged by a dynamo, while the other is doing the active work of, say, lighting a series of lamps. When the first one is completely charged, and the decomposition of gas has begun, the gas given off operates an automatic switch, and shunts the charging current on to the battery which is nearly exhausted, while the one which is completely charged is by the same movement switched into the discharging circuit. His claims cover the use of the gases thus given off, and for this purpose, and the whole general arrangement, as above.

W. L. Voelkler has patented (251,748) a device for preventing waste ef energy in a circuit of, say, electric lights. He introduces a series of secondary batteries so automatically connected that all energy given off by the dynamo above a certain and necessary amount is stored up in them; whereas, if for any reason the energy of the generator falls below the required amount, the force stored up in these cells is at once brought into play, and thus the current is kept constant. This is accomplished by means of a strip of metal running over an insulated pulley and loaded with a weight, which plunges into a reservoir of mercury. The metal strip and mercury are part of the main circuit. Excess of energy heats the strip and elongates it, allowing the weight to descend and displace the quicksilver, which rises in another tube, and makes contacts which switch off part of the current into the secondary batteries. On the other hand, if the metal strip becomes too cool, it, by a reverse action, throws the secondary batteries into the main circuit.

H. B. Sheridan has another device (253,435) for a similar purpose, but particularly intended for lighting railway cars. The dynamo is connected with the running-gear. He inserts an automatic switch, whereby the main circuit is broken when the charging current becomes weak, or stops, in order to prevent the current passing back from the storage cells to the generator. Instead of a strip of metal, as in the previous system, he has a series of lever switches actuated by cores, drawn into magnet helices, as the strength of the current varies.

C. E. Buell patents (255,248) a mode of preventing waste of the charge of a secondary battery, when the generator, which charges it, is either at rest or in slow action. Were this not prevented, the energy accumulated in the storage box might return to the generator and be virtually lost. He has a developing and a charging circuit. When the dynamo is first started up, electrical power is quickly developed, and when sufficient speed is attained the current is turned off, either automatically or by hand, and on to the charging circuit.

Buell also patents a scheme something like this (255,249) : A charging circuit, two series of secondary batteries, a working circuit common to both, a switching device for alternately connecting one series in the charging circuit, the other in the working circuit, and an arrangement by which the series being charged, are *coupled for quantity*, the series being discharged for *intensity*.

In still another patent (259,362), Buell makes this combination quite effective for lighting cars, each car being provided with switching devices, and two series of secondary batteries, those being charged being coupled for quantity, and those working the lights, for intensity. Moreover, the storage boxes may be thrown entirely out of the circuit, and the charging current made to do the work of lighting.

In conclusion, then, secondary batteries are not universally applicable, but within certain limits their value will prove—in the near future—very great. As regulators and equalizers of powerful currents, in numerous domestic and professional applications, and as a means of economizing in the first cost of large plants they are without a visible rival. For instance, a one-horse power dynamo, intended to run for twelve hours, in storing up a charge, is much less expensive than a twelve-horse power machine, intended only to run an hour, while, in many cases, running the former will be found little or no more expense than the latter. In Paris, if the writer remembers aright, and in other cities, a company will charge up small accumulators for an evening's lighting, calling for them next day, and leaving freshly charged ones, and for a very small sum.

The patents which I have summarized in this paper will serve to give an idea of the number of services to which secondary batteries may be put. We must remember that, as applied for commercial purposes, this matter is still in its infancy, and that great improvements and additions may be expected in its scientific front. The little instrument with which I show you a few results to-night is as a scientific plaything, when compared with the immense surface of lead offered in some of the polarization batteries now in actual use for electric motors and lights.

In all that I have said to-night, I feel that I have not been able to do justice to the industrious talent of Planté and others (to whom all honor), nor to throw that electric light upon the subject which it deserves; but I venture to hope that I have shown that it is one which has, literally, a most brilliant future.

1558 WABASH AVE.

